# The Effect of Antenatal Paracetamol on Breathing Effort of Premature Infants at Birth: A Feasibility Trial

**DOI:** 10.3390/children13081061

**Published:** 2026-08-10

**Authors:** Timothy J. R. Panneflek, Janneke Dekker, Kristel L. A. M. Kuypers, Douglas P. Derleth, Graeme R. Polglase, Lotte E. van der Meeren, Merlijn Wind, Remco Visser, Stuart B. Hooper, Thomas van den Akker, Arjan B. te Pas

**Affiliations:** 1 Division of Neonatology, Department of Paediatrics, Willem-Alexander Children’s Hospital, Leiden University Medical Centre, 2333 ZA Leiden, The Netherlands; 2 Department of Paediatric and Adolescent Medicine, Mayo Clinic College of Medicine, Rochester, MN 55905, USA; 3 The Ritchie Centre, Hudson Institute of Medical Research, Melbourne, VIC 3168, Australia; 4 Department of Obstetrics and Gynaecology, Monash University, Melbourne, VIC 3168, Australia; 5 Department of Pathology, Leiden University Medical Centre, 2333 ZA Leiden, The Netherlands; 6 Department of Pathology, Erasmus Medical Centre, 3000 CA Rotterdam, The Netherlands; 7 Department of Obstetrics and Gynaecology, Leiden University Medical Centre, 2333 ZA Leiden, The Netherlands; 8 Athena Institute, VU University, 1081 HV Amsterdam, The Netherlands

**Keywords:** breathing, resuscitation, neonatology, paracetamol, premature infants

## Abstract

**Highlights:**

**What are the main findings?**

• Antenatal paracetamol administration 30–120 min prior to birth is feasible in only half of women giving birth <31 weeks’ gestation, which is below the pre-specified feasibility threshold of 80%.
• Antenatal paracetamol may potentially increase spontaneous breathing in premature infants at birth.

**What is the implication of the main finding?**

• This feasibility trial provides a rationale and framework for larger studies to assess the effect of antenatal paracetamol on respiratory outcomes for premature infants at birth.

**Abstract:**

**Background:** Prostaglandin E_2_ (PGE_2_) suppresses perinatal breathing. Reducing antenatal PGE_2_ concentrations with paracetamol could stimulate spontaneous breathing in premature infants at birth. We aimed to assess feasibility and potential effects of antenatal paracetamol. **Methods:** In this randomised, placebo-controlled, triple-blind feasibility trial, pregnant women <31 weeks’ gestation were randomised to receive either intravenous paracetamol (intervention) or placebo (control). Multiple pregnancies were also eligible, and the number of infants could exceed the number of enrolled women. The primary outcome was feasibility of administering study medication within 30–120 min prior to birth. Secondary exploratory outcomes included measures of breathing and other physiological parameters of infants during the first 1–10 min after birth and were compared between infants from mothers who received paracetamol and infants from mothers who received a placebo irrespective of timing of administration. Planned enrolment was 40 women. **Results:** The trial enrolled 23 women before termination due to low recruitment. Five women did not receive study medication, while 18 did (9 per group). Overall, 11 of the 23 enrolled women (48%) received study medication within the specified timeframe. The nine women in the intervention group gave birth to 13 infants, while the nine women in the control group gave birth to 12 infants. Among infants, average minute volume did not differ significantly between groups (intervention (*n* = 13) vs. control (*n* = 12); mean ± SEM; 147 ± 12 vs. 114 ± 14 mL/kg/min, *p* = 0.093). However, the intervention group had a significantly higher area under the curve for minute volume, greater inspiratory drive, and a higher heart rate (1120 ± 410 vs. 707 ± 346 mL/kg, *p* = 0.020; 11.8 ± 1.0 vs. 8.4 ± 1.3 mL/kg/breath/s, *p* = 0.046; 137 ± 5 vs. 121 ± 5 beats/min, *p* = 0.037). **Conclusions:** Antenatal paracetamol administration 30–120 min prior to birth was feasible in only half of the women, below the pre-specified threshold of 80%, but the exploratory findings suggest that it may improve the breathing effort of premature infants.

## 1. Introduction

Premature infants have an immature respiratory system and often require respiratory support to aerate their lungs and establish gas exchange [1]. As respiratory support is provided non-invasively, its effectiveness is dependent on an infant’s spontaneous breathing effort [1]. Spontaneous breathing ensures laryngeal patency, which is necessary for the air and oxygen provided during respiratory support to reach the lungs [2,3]. For non-invasive respiratory support to be effective at birth, emphasis should be placed on stimulation of spontaneous breathing [1].

Spontaneous breathing at birth is influenced by many factors, including physical stimuli, the infant’s arousal state, oxygenation and inflammation [1]. Inflammation is thought to depress breathing by the release of inflammatory mediators, such as prostaglandins [4]. Prostaglandins, particularly prostaglandin E_2_ (PGE_2_), suppress respiratory drive generated in the brainstem by binding to receptors that lower neuronal firing rate [5]. Premature infants are at risk of depressed spontaneous breathing at birth, because a large proportion of them are exposed to events—such as preterm labour, hypoxia and antenatal inflammation in the form of chorioamnionitis—that increase antenatal prostaglandin exposure [6,7,8,9].

Reducing antenatal prostaglandin concentrations could potentially stimulate spontaneous breathing in premature infants. Paracetamol, an acetanilide derivative, is known to decrease central PGE_2_ concentrations by various mechanisms [10]. In addition, paracetamol crosses the placenta and has been shown to stimulate foetal breathing movements in lambs [11,12,13]. Moreover, a retrospective observational matched cohort study has demonstrated that maternal oral antenatal paracetamol administration <24 h prior to birth was associated with fewer acute rescue interventions in premature infants at birth, particularly a reduced surfactant requirement [14].

Paracetamol pharmacokinetics change during pregnancy. As pregnancy advances, the volume of distribution and clearance increase. This lowers paracetamol concentrations and shortens the half-life of paracetamol [15,16,17,18]. In order for a paracetamol dose (maximum of 1 g) to achieve concentrations within its therapeutic range (i.e., >10 mg/L for pain management), we considered that it should be administered intravenously within 30–120 min prior to birth [10]. Therefore, we primarily investigated whether it was feasible to administer paracetamol within 30–120 min prior to birth and secondarily whether this could improve spontaneous breathing in premature infants at birth.

## 2. Materials and Methods

This single-centre, triple-blind, randomised and placebo-controlled drug feasibility trial (registered before subject recruitment as EudraCT 2022-003415-29) was conducted between September 2023 and January 2025 at the Leiden University Medical Centre (LUMC), a tertiary-care centre in the Netherlands. The trial was approved by the Central Committee on Research Involving Human Subjects (CCMO; NL82714.000.22), serving as the Institutional Review Board. The Ministry of Health, Welfare and Sport carried out the marginal review.

All participating parties (i.e., researchers, obstetricians, neonatal staff and expectant parents) were blinded to treatment allocation except for the obstetric nurses who prepared and administered the study medication. The obstetric nurses had no interactions with the prescribers of the study medication to ensure concealment of allocation. Researchers were unblinded only after data analyses had finished.

Initially, only pregnant women between 24^+0^ and 29^+6^ weeks’ gestation were eligible for inclusion. Following a protocol amendment in May 2024, pregnant women between 30^+0^ and 30^+6^ weeks’ gestation were also included. This was done to increase enrolment rates and reach the target sample size, given the slower-than-anticipated participant recruitment (i.e., only 10 inclusions in the first 8 months after the start of the study). Antenatal informed consent was necessary for participation in the trial. Exclusion criteria are listed in the Supplemental File.

Parents were not approached for study participation if there was a language barrier (insufficient Dutch or English), as informed consent could not be adequately ensured; if there was insufficient time between hospital admission and birth to acquire antenatal informed consent; or if acquiring informed consent was considered inappropriate by the attending physician.

Pregnant women admitted to the maternity ward who consented to participate in the study were randomised to either the paracetamol or placebo group after the attending physician, under the supervision of an obstetrician (hereafter obstetrician), predicted that birth would occur within 30–120 min. For this study, we compiled a list of criteria to create a uniform assessment of expected birth. This list comprised: (A) end of the first stage of labour in nulliparous women, (B) beginning of active labour in multiparous women and (C) one hour prior to the anticipated start of a caesarean section. Ultimately, it was up to the discretion of the obstetrician when to administer the study medication. Randomisation occurred 1:1 in blocks of four prior to the study by the trial pharmacy of the LUMC. The study kits were randomised prior to the start of the study and then allocated to pregnant women based on their gestation: under or above 28 weeks’ gestation. All pregnant women with a threatening preterm birth below 32 weeks’ gestation were recommended to receive antenatal magnesium infusion for foetal neuroprotection according to the local protocol.

Pregnant women randomised to the intervention group received 1000 mg of paracetamol intravenously (IV; 100 mL Fresenius Kabi RVG 105747 10 mg/mL solution containing mannitol, cysteine and water; Fresenius Kabi Nederland B.V., Huis ter Heide, The Netherlands) via an existing infusion line. Pregnant women randomised to the control group received IV-placebo (100 mL Fresenius Kabi 0.9% NaCl concentrate; Fresenius Kabi Nederland B.V., Huis ter Heide, The Netherlands). An additional dose of study medication was administered if labour did not occur within the designated timeframe, with a maximum of three dosages and a minimum of 4–6 h between dosages. Placentae were sent to the pathology department for determination of antenatal inflammation as defined by the Amsterdam Placental Workshop Consensus statement [19].

Pregnant women filled in a questionnaire detailing (timing of) previous paracetamol use during pregnancy. All obstetricians that prescribed study medication were asked to fill in an online survey detailing their experiences with the study. The survey included questions about use of our compiled criteria list for study medication administration, feasibility, workload, obstacles and possible improvements in the study.

The study was classified as a negligible-risk study and a clinical trial with limited interventions; therefore, a Data Safety Monitoring Board was not required. The classification was based on prior evidence demonstrating the safety of intravenous administration of 1000 mg paracetamol prior to birth [20,21,22,23,24,25]. In addition, the trial was conducted in accordance with European Medicine Agency (EMA) and U.S. Food and Drug Administration (FDA) recommendations for paracetamol administration. Monitoring was performed twice during the study period by an accredited monitor, with no major concerns identified. No adverse event, serious adverse events, or suspected unexpected serious adverse reactions were observed.

At birth, infants were stabilised according to local protocol (Supplemental File). Cord clamping occurred between 30 and 60 s after birth and respiratory support was commenced on a standard resuscitation table until April 2024. After April 2024, infants born vaginally received physiological-based cord clamping using the Concord table [26]. A respiratory function monitor (Advanced Life Diagnostics resuscitation monitor, Weener, Germany) was used to visualise pressures and delivered tidal volumes.

Oxygen saturation (SpO_2_) and heart rate (HR) were measured with a Masimo SET pulse oximeter positioned on the right hand of the infant (Masimo Radical, Masimo Corporation, Irvine, CA, USA). The fraction of inspired oxygen (FiO_2_) was measured with a portable oxygen analyzer AX300-I (Teledyne Analytical Instruments, City of Industry, CA, USA), and the inspired and expired flow was measured using a variable orifice flow sensor (Avea Varflex Flow Transducer, Carefusion, Yorba Linda, CA, USA). All signals were recorded electronically using the NewLifeBox-R physiological recording system (Advanced Life Diagnostics, Weener, Germany) coupled with the Polybench physiological software (Applied Biosignals, Weener, Germany). Pulmochart software (Applied BioSignals, Weener, Germany) was used for a breath-by-breath analysis corrected for birthweight.

The primary outcome of this study was feasibility, which was defined as the success rate of pregnant women receiving three or fewer doses of the study medication, within 30 to 120 min prior to birth at a gestation of <31 weeks. In the case of a multiple pregnancy, only one infant needed to be born within the specified timeframe for it to be labelled as a success. Additional feasibility parameters included: latency period between administered medication and birth and obstetrical survey questions (use of our compiled criteria list, feasibility, workload, obstacles and possible improvements in the study).

The secondary exploratory outcome was breathing effort, assessed as the average minute volume (mL/kg/min) measured 1–10 min after birth and the area under the curve for minute volume (mL/kg) in this period. Area under the curve represents the cumulative minute volume over time, indicating the total volume of air inhaled during the measured period. Breathing effort was the product of inspiratory tidal volume (mL/kg/breath) and respiratory rate (breaths/min). Inspiratory tidal volume was measured during spontaneous breathing on CPAP with a mask leak <75% (mL/kg/breath), because a mask leak > 75% in the delivery room had previously been considered as clinically relevant [27]. Respiratory rate was assessed independent of the type of respiratory support used. Additional physiological parameters assessed in the first 1–10 min after birth included: inter-breath variability (expressed as coefficient of variation (COV; %)), inspiratory drive (mL/breath/kg/s), a reflection of phrenic nerve activity [28,29], apnoea (cessation of breathing ≥ 10 s), HR (beats/min), SpO_2_ (%) and FiO_2_ (%).

Maternal and neonatal baseline characteristics consisted of: maternal age, parity, gestational age, multiple pregnancy, mode of birth, general anaesthesia, antenatal corticosteroids, opioids, clinical chorioamnionitis [30], histological chorioamnionitis [19], birthweight, sex, umbilical pH, physiological-based cord clamping, and Apgar scores at 1 and 5 min after birth.

### Statistical Analysis

To determine feasibility, we aimed to have a success rate of 80% of pregnant women receiving study medication within 30–120 min prior to birth. Previous delivery room trials have also determined feasibility as successful implementation of the study intervention in 80% of participants [31]. We believed that this would reflect an acceptable threshold for assessing practical applicability. The threshold was defined a priori to objectively assess the feasibility of the intervention.

The sample size was derived from the primary outcome (feasibility) rather than the secondary exploratory outcomes (breathing effort), as this was a feasibility trial. The reference range of sample sizes in feasibility studies is variable, but the interquartile range is between 21 and 43 [32]. This indicates that a sample size of 21 is sufficient to assess feasibility and enrolment beyond this minimum primarily increases the precision of the estimate. Accordingly, we aimed to include 40 pregnant women: 20 in the paracetamol group and 20 in the placebo group. This would also enable a preliminary comparison between the groups.

The study was initially expected to take only nine months. After seventeen months, the study was stopped prematurely in January 2025 after including 23 pregnant women due a low enrolment rate.

Statistical analyses were performed with IBM SPSS Statistics V.29.0 (IBM Corp., Armonk, NY, USA, 2022). Graphics were made using GraphPad Prism (v.9).

Continuous data was assessed for normality by inspection of histograms in addition to the Shapiro–Wilk test, and in cases of repeated measures, individual data points were assessed for normality. Continuous variables were presented as median (IQR) or mean ± standard deviation, categorical variables as *n* (%), and open-ended questions as a general summary. Missing data is noted in the corresponding table.

The primary study outcome of feasibility is described as *n* (%). The secondary exploratory outcome of breathing effort was compared between infants in the intervention group receiving IV-paracetamol and those in the control group receiving IV-placebo. Breathing effort was compared with two analyses. First, to assess the effect of paracetamol on breathing effort over time while accounting for repeated measurements within infants, we used a linear mixed model with a first-order autoregressive variance covariance matrix that imputed missing data. Fixed effects in the regression analysis comprised time, treatment and time*treatment. The *p*-value for the treatment covariate is the main outcome for breathing effort, whereas the *p*-value for time*treatment informs whether groups differed over time. Time is marked in minutes and allows for a maximum of 9 data points per study participant. The outcome of the linear mixed-effects model was described as estimated marginal mean ± standard error of the mean (SEM) of the treatment fixed effect. Second, to evaluate the effect of paracetamol on cumulative inspired volume, we compared the area under the curve of minute volume as a single summary measure between infants in the intervention and control groups using an Independent-Samples Student’s *t*-test.

All respiratory and additional physiological outcomes were compared between the infants of the intervention and control groups over time with a linear mixed model identical to the one described for breathing effort. Other study parameters were compared between groups with Independent-Samples Student’s *t*-tests for parametric data, Mann–Whitney U tests for non-parametric data and Pearson Chi^2^ tests or Fisher’s exact tests for categorical data. The corresponding tests are reported in the tables.

A two-sided *p*-value < 0.05 is considered statistically significant. We did not correct for multiple testing or clustering of infants from multiple pregnancies, as this feasibility trial was hypothesis-generating. Results are exploratory and intended to inform the design and power of subsequent definitive trials.

## 3. Results

**Figure 1:**
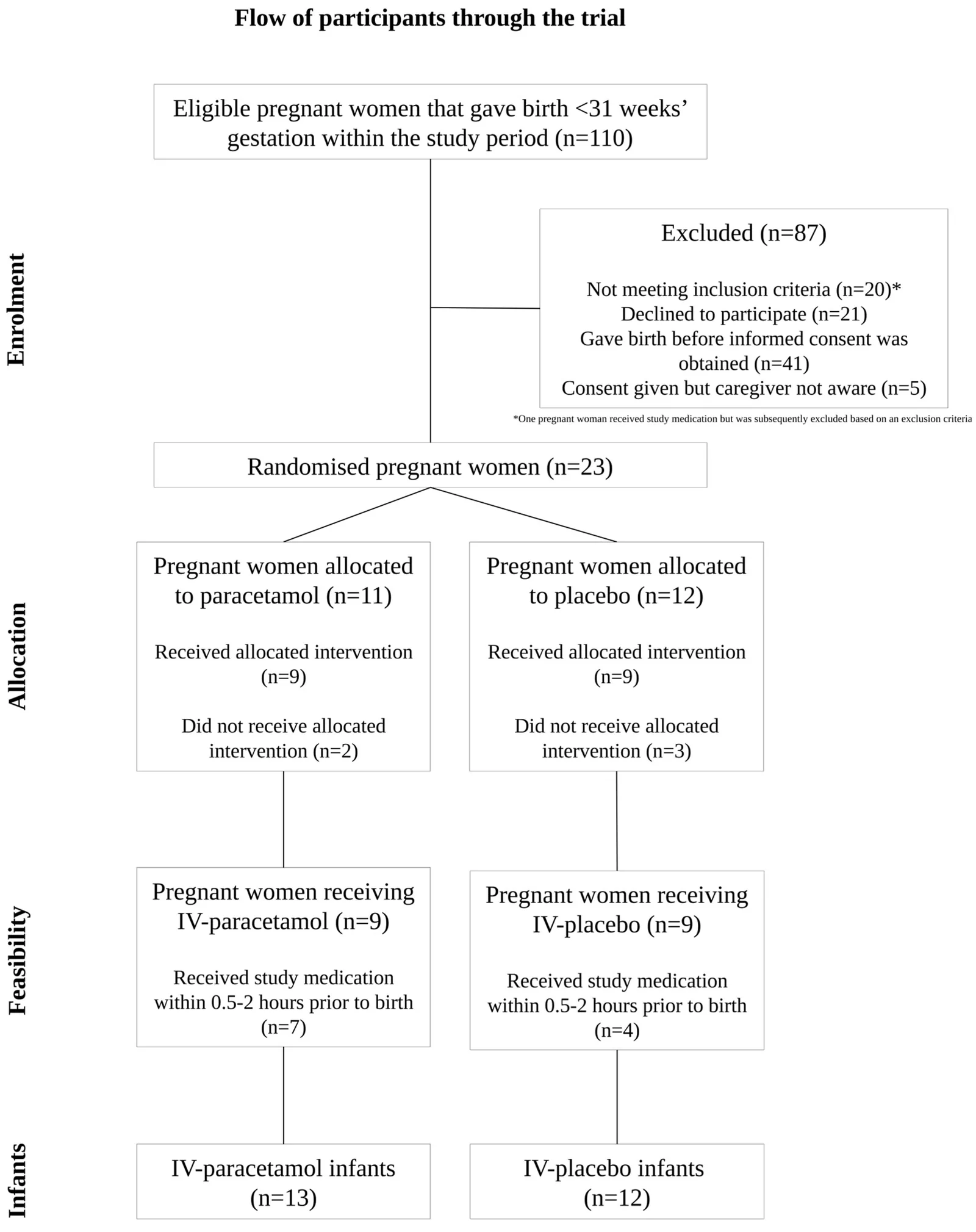
Flow diagram of the study protocol. CONSORT flow diagram of participants (pregnant women and infants) during the study period.

A total of 110 pregnant women gave birth between 23^+0^ and 30^+6^ weeks’ gestation during the study period, of whom 90 were eligible for inclusion (Figure 1). Forty-one women gave birth before informed consent could be obtained. Of the remaining 49 women, 28 (57%) gave informed consent. Of these, five women were not included because the attending physicians were unaware of their consent. In total, 23 women (16 single and 7 multiple pregnancies) were included, who gave birth to a total of 31 infants.

### 3.1. Baseline Characteristics

Of the 23 women included in the study, nine women received IV-paracetamol, nine women received IV-placebo, and five women did not receive study medication (Figure 1). One woman in the paracetamol group and three women in the placebo group received more than one dose of study medication. The nine women receiving paracetamol gave birth to 13 infants, and the nine women receiving placebo gave birth to 12 infants. Maternal and neonatal characteristics were similar between groups (Table 1).

### 3.2. Feasibility

**Figure 2:**
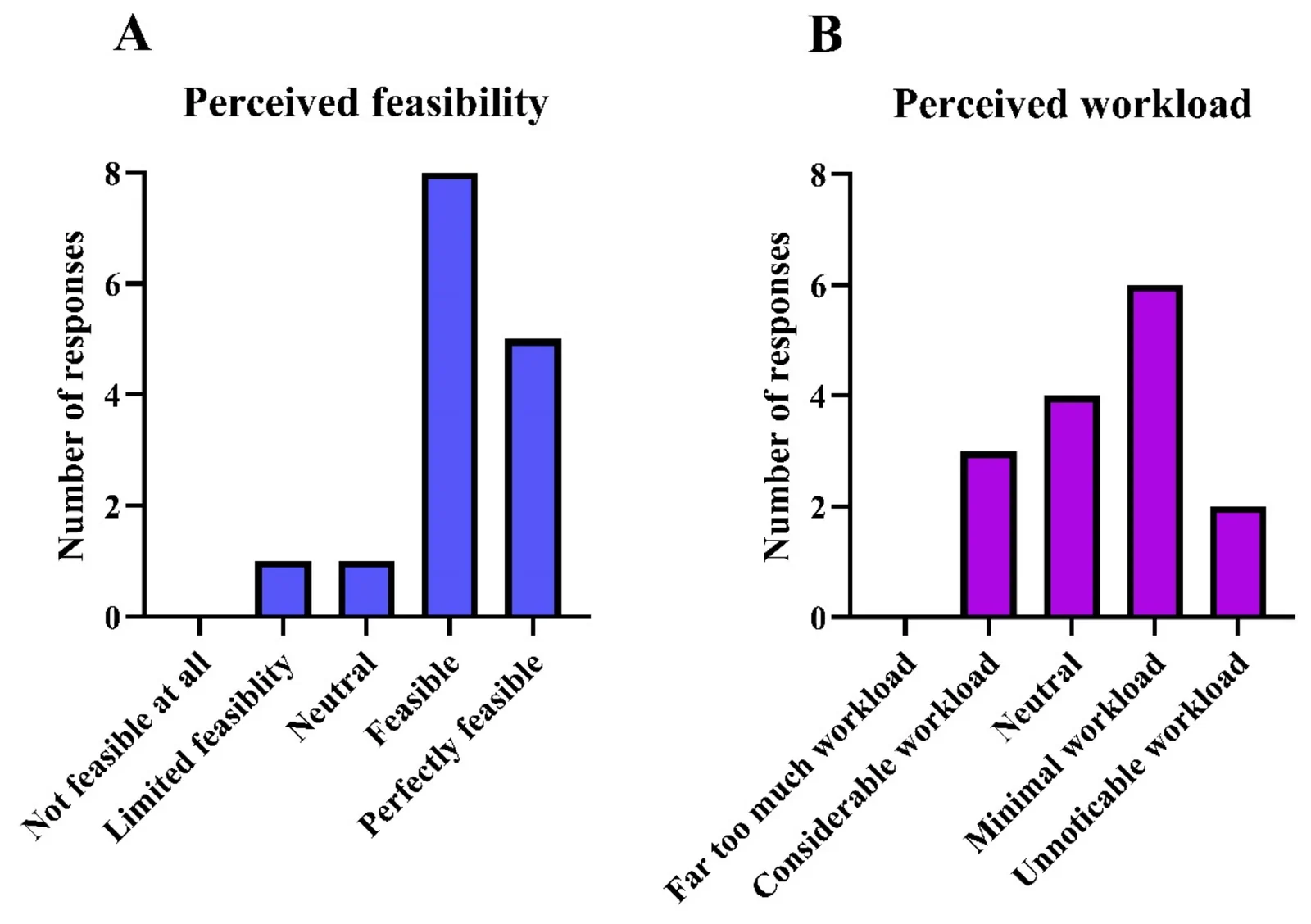
Survey results. Evaluation of the (**A**) feasibility and (**B**) workload of the study protocol by obstetricians (in training).

Of the 23 included women, five women (22%) did not receive study medication due to: birth before study medication could be given (*n* = 2) and logistical issues (*n* = 3). Eleven of the twenty-three pregnant women (48%) received the study medication within 30–120 min prior to birth: 5/13 (38%) vaginal births and 6/10 (60%) caesarean sections. Median (IQR) time of administration was 70 (35–137) minutes before birth. Fifteen of the twenty-three obstetricians who assisted with the births (65%) filled in the survey. According to the obstetricians, 75% succeeded in administering the study medication in the correct timeframe and 81% used the provided protocol. Evaluation of feasibility and workload of the protocol are displayed in Figure 2. Reported obstacles included: timely administration of medication, inclusion rate, workload for nurses, and study protocol training of nurses. Reported possible improvements included: training of nurses, larger administration windows, implementation of treatment as standard care, and providing more reminders to increase study awareness.

### 3.3. Breathing Effort of Infants

**Figure 3:**
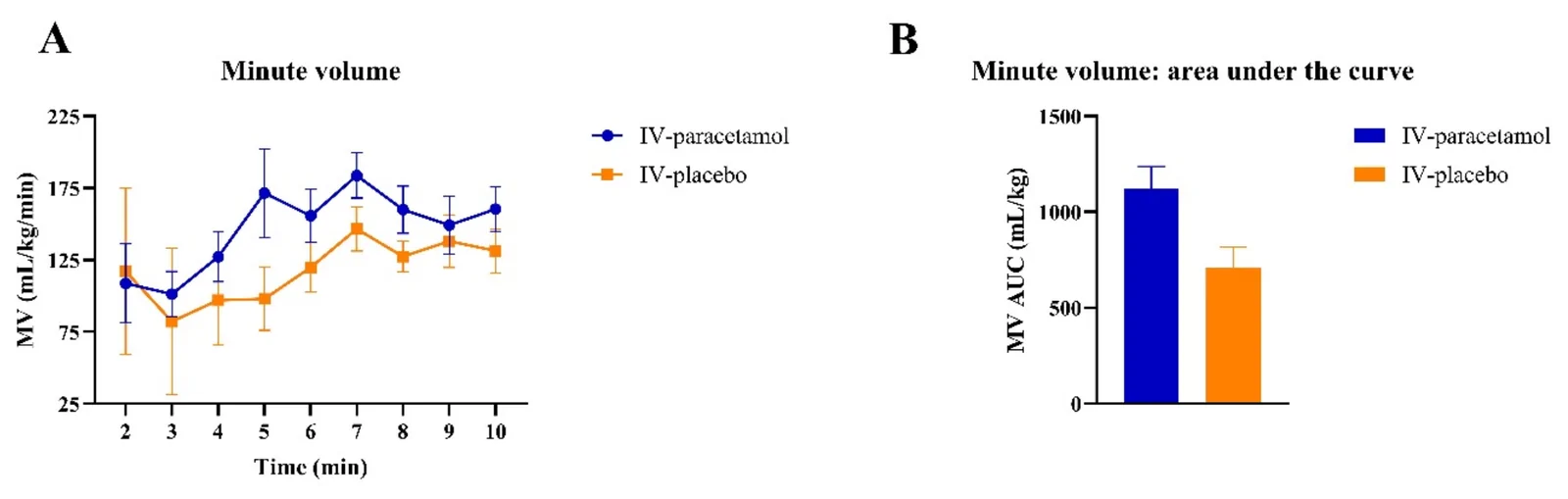
Minute volume in the first 1–10 min after birth. (**A**) Minute volume (MV) in infants (*n* = 13; blue) randomised to the intervention group, antenatal IV-paracetamol, and infants (*n* = 12; orange) randomised to the control group, antenatal IV-placebo, in the first 1–10 min after birth. (**B**) Area under the curve (AUC) of MV in infants (*n* = 13; blue) randomised to antenatal IV-paracetamol and infants (*n* = 12; orange) randomised to antenatal IV-placebo in the first 1–10 min after birth. Data is presented as mean ± standard error of the mean (SEM).

**Figure 4:**
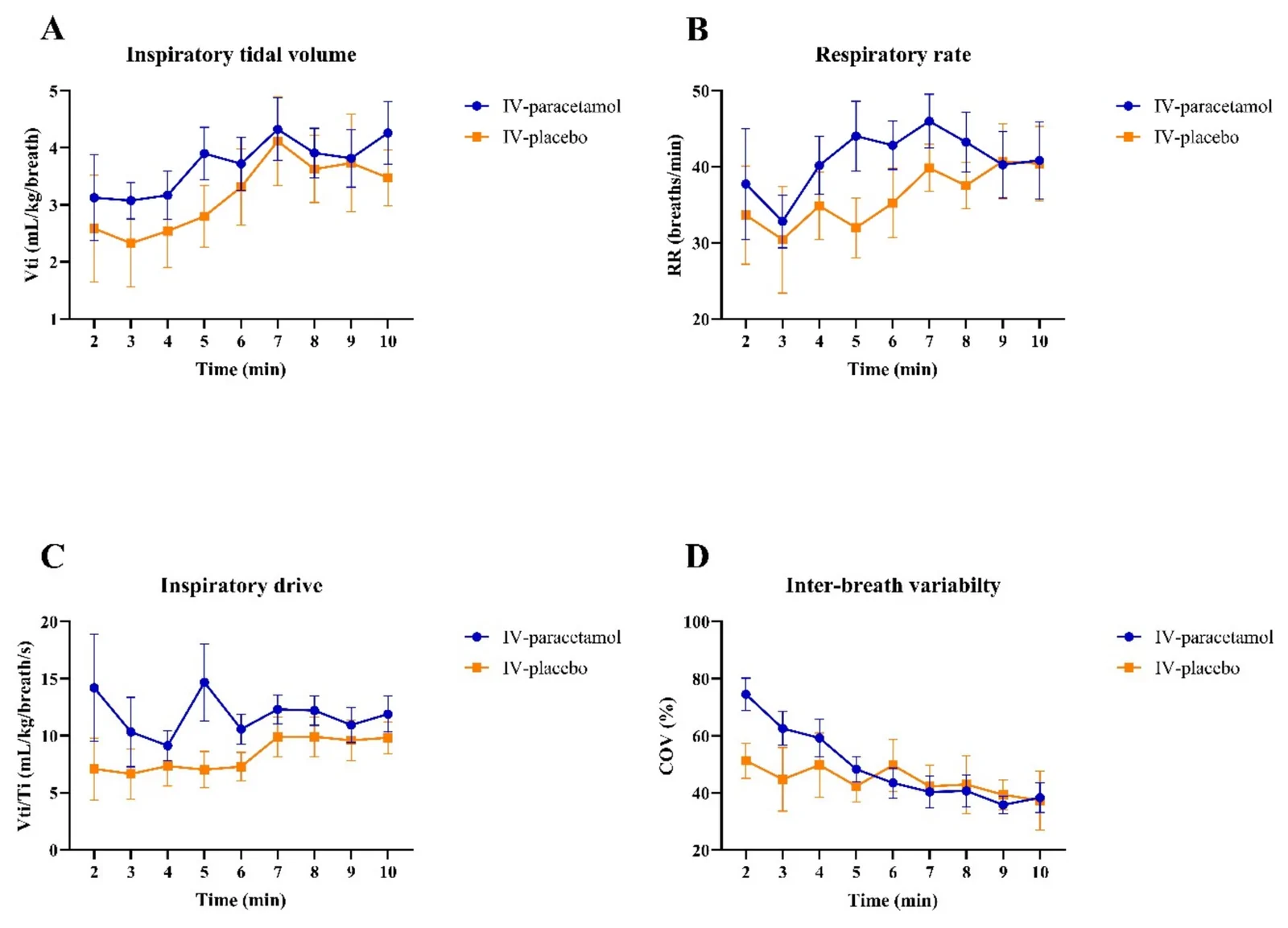
Respiratory parameters in the first 1–10 min after birth. (**A**) Inspiratory tidal volume (Vti), (**B**) respiratory rate (RR), (**C**) inspiratory drive (Vti/Ti), and (**D**) inter-breath coefficient in variability (COV) in infants (*n* = 13) randomised to the intervention group, antenatal IV-paracetamol, and infants (*n* = 12) randomised to the control group, antenatal IV-placebo, in the first 1–10 min after birth. Data is presented as mean ± standard error of the mean (SEM).

Among the 25 included infants whose mothers received study medication, average minute volume during the first 10 min after birth was not significantly higher in the intervention group compared to the control group (intervention group (*n* = 13) vs. control group (*n* = 12); mean ± SEM; 147 ± 12 vs. 114 ± 14 mL/kg/min, *p* = 0.093) (Figure 3), but the area under the curve for minute volume was significantly higher in the intervention group (1120 ± 410 vs. 707 ± 346 mL/kg, *p* = 0.020) (Figure 3). No differences between groups in other respiratory parameters (over time) were found, except for higher inspiratory drive in the intervention group (11.8 ± 1.0 vs. 8.4 ± 1.3 mL/kg/breath/s, *p* = 0.046) (Table 2 and Figure 4).

### 3.4. Additional Physiological Parameters

**Figure 5:**
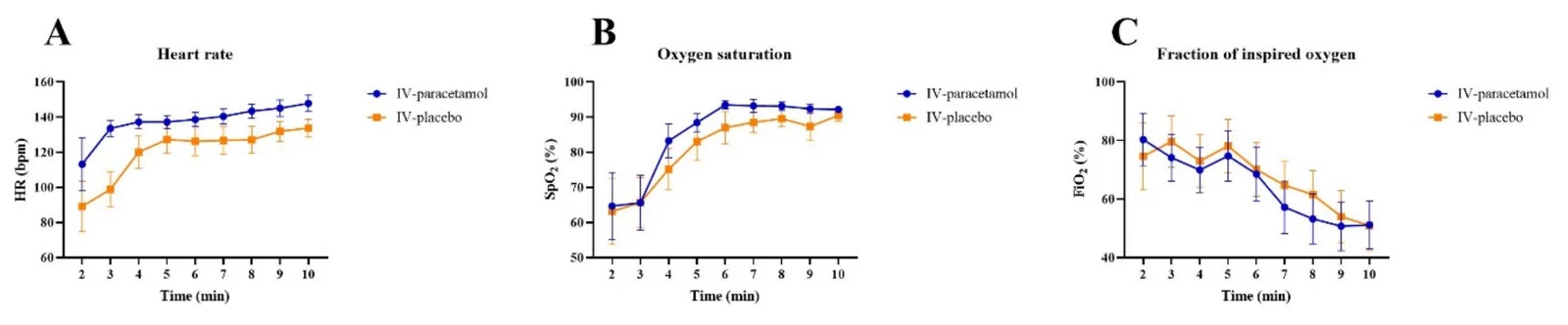
Additional physiological parameters in the first 1–10 min over time. (**A**) Heart rate (HR), (**B**) oxygen saturation (SpO_2_), and (**C**) fraction of inspired oxygen (FiO_2_) in infants (*n* = 13) randomised to the intervention group, antenatal IV-paracetamol, and infants (*n* = 12) randomised to the control group, antenatal IV-placebo, in the first 1–10 min after birth. Data is presented as mean ± standard error of the mean (SEM).

Infants in the intervention group had a significantly higher HR compared to infants in the control group (137 ± 5 vs. 121 ± 5 beats/min, *p* = 0.037), and they reached HR >100 beats/minute faster (median (IQR); 2:10 (1:41–2:25) vs. 2:51 (2:31–3:13) min, *p* = 0.019) (Figure 5 and Table 2). There were no differences between groups in SpO_2_ and FiO_2_, or in changes over time for HR, SpO_2_ and FiO_2_ (Figure 5 and Table 2).

### 3.5. Respiratory Support Outcomes

There were no differences between groups in respiratory support provided in the delivery room or the NICU, and the initial rectal temperature in the NICU was similar (mean ± SD; 36.6 ± 0.6 vs. 36.5 ± 0.6 (°C), *p* = 0.830) (Table 3).

## 4. Discussion

This randomised, controlled feasibility trial failed to reach its target sample size after enrolling 23 women, but demonstrated that administration of paracetamol 30–120 min prior to premature birth was feasible in only 48% of women, which was below the pre-specified feasibility threshold of 80%. In addition, timely administration was feasible in only 38% of all vaginal births and 60% of caesarean sections. In the exploratory secondary findings, we observed that average minute volume in the first 10 min after birth was not significantly different between premature infants of mothers treated with IV-paracetamol (intervention group) and infants of placebo-treated mothers (control group). However, cumulative minute volume (area under the curve) was higher in the intervention group in addition to inspiratory drive, a reflection of the level of respiratory-centre stimulatory activity, and heart rate, suggesting a potential benefit of antenatal paracetamol.

This feasibility trial was designed to evaluate s protocol intended for a larger study. We demonstrated that the current approach was not feasible due to the study design and the time constraints for paracetamol administration. The requirement for antenatal consent and the narrow administration window introduced substantial logistical challenges. Most eligible women could not be approached before giving birth and the consent rate was 57%, limiting enrolment. Nevertheless, this study exceeded the minimum sample size required to assess feasibility (*n* = 21) [32] and for between-group comparisons in feasibility trials (*n* = 12 per group) [33], thereby allowing a reliable evaluation of feasibility and hypothesis-generating exploratory findings.

Feasibility of timely study medication administration was low: 22% of enrolled women did not receive study medication and less than half received study medication within the intended time window. Feasibility was lower in vaginal birth (38%) than in caesarean sections (60%), underscoring the difficulty of predicting the timing of preterm birth. These findings identify key areas for protocol refinement in a future larger study.

Our findings align with previous studies. Regarding safety, multiple randomised clinical trials investigating the effect of 1000 mg intrapartum paracetamol reported no maternal, foetal or neonatal adverse events, consistent with our results [20,21,22,23,24,25]. Prospective cohort studies also suggest negligible cardiovascular risk from paracetamol exposure in any trimester [34]. Furthermore, a meta-analysis of observational studies found no association between early postnatal paracetamol exposure and neurodevelopmental complications [35]. Similar findings were reported in a retrospective cohort study of premature infants [36], providing reassurance regarding paracetamol exposure close to birth, as investigated in our study. Regarding effectiveness, experimental studies in foetal lambs show that IV-paracetamol increases both the rate and depth of foetal breathing movements while lowering circulating PGE_2_ concentrations [13]. Clinical observational studies have demonstrated that (i) prenatal paracetamol exposure is associated with fewer rescue interventions (mainly surfactant administration) at birth [14], (ii) prenatal paracetamol exposure correlates positively with Apgar scores [14], and (iii) paracetamol intake by infants with bronchiolitis is protective against apnoea [37].

Our exploratory and hypothesis-generating secondary findings suggest a potential effect of antenatal paracetamol on spontaneous breathing. Although average minute volume during the first 10 min after birth was not significantly different between groups, infants receiving IV-paracetamol demonstrated a greater cumulative minute volume, reflected by a higher area under the curve. This indicates that, while minute volume at individual time points was not consistently higher due to paracetamol, the total volume of air inhaled during the first 10 min was greater. Interestingly, infants receiving IV-paracetamol also exhibited a higher heart rate. This may reflect accelerated lung aeration, as lung aeration triggers an increase in both pulmonary blood flow and pulmonary venous return, thereby elevating cardiac output and heart rate [38]. Since spontaneous breathing drives lung aeration, these physiological findings may be consistent with a possible increase in respiratory drive following antenatal paracetamol, though it is unclear whether this also translates into improved clinical outcomes, such as reduced need for intubation or better long-term prognosis.

The present study proposed that lowering PGE_2_ concentrations with antenatal paracetamol could stimulate spontaneous breathing. However, we did not measure paracetamol or PGE_2_ concentrations in premature infants at birth. As a result, we are unable to substantiate the biological plausibility of our findings, nor define the therapeutic range of paracetamol, or the reduction in plasma PGE_2_ concentrations required to stimulate breathing activity in premature infants at birth. However, perinatal circulating PGE_2_ concentrations are known to be very variable [39], especially in the presence of antenatal inflammation [40]. In addition, paracetamol concentrations are also likely to vary considerably due to changes in maternal pharmacokinetics throughout pregnancy [15,16,17,18], as well as the range of gestational ages and body mass indices in the study. In this small sample, such variability is likely to reduce the added value of pharmacokinetic and pharmacodynamic analyses.

In the present study, we enrolled fewer participants than planned and performed multiple exploratory analyses without adjustments for multiple testing or clustering of infants from multiple pregnancies, mainly increasing the risk of Type-I errors (i.e., false-positive results). Accordingly, these exploratory findings should be considered hypothesis-generating and warrant confirmation in larger studies.

A potential next step is the implementation of intravenous paracetamol just prior to birth to study its effects in a pre–post implementation study. This design could enhance the feasibility of antenatal paracetamol administration within 30–120 min prior to birth by removing some of the logistical barriers, such as training of nurses and blinded study medication preparation. Furthermore, as feasibility needs to be improved for practical application, physicians may become more adept at timely administration through a positive learning curve, as observed in other large delivery room trials [26]. Moreover, a pre–post implementation study does not require antenatal informed consent, which may facilitate higher inclusion rates, increasing the statistical power of the study. Considering the global availability of paracetamol and the high annual incidence of preterm birth affecting millions of infants, paracetamol may be a simple and scalable intervention to improve respiratory and other clinical outcomes in premature infants worldwide.

## 5. Conclusions

In this randomised, placebo-controlled, triple-blind feasibility trial, antenatal administration of paracetamol between 30 and 120 min prior to birth was feasible in only half of the pregnant women included in the study, which was below the pre-specified feasibility threshold of 80%. While antenatal paracetamol may increase spontaneous breathing in premature infants, the small sample size precludes definitive conclusions regarding these exploratory effects. Larger studies with an improved design are warranted to assess the role of antenatal paracetamol in supporting the cardiopulmonary transition at birth.

## Figures and Tables

**Table 1:** Baseline characteristics.

	IV-Paracetamol (*n* = 9)	IV-Placebo (*n* = 9)	*p*-Value
**Maternal** **baseline characteristics**			
Maternal age (yrs)	30 (29–34)	30 (29–39)	0.625 ^a^
Primiparity	7 (78%)	6 (67%)	0.599 ^b^
Multiple gestation	4 (44%)	2 (22%)	0.317 ^b^
Body mass index (kg/m^2^)	30 ± 7	31 ± 7	0.729 ^c^
Premature prelabour rupture of membranes	2 (22%)	2 (22%)	1.000 ^b^
Earlier use of paracetamol during pregnancy	6 (75%)	6 (75%)	1.000 ^b^
Full course of antenatal corticosteroids	9 (100%)	8 (89%)	1.000 ^d^
Opioids	1 (11%)	2 (22%)	0.527 ^b^
Caesarean section	4 (44%)	3 (33%)	0.629 ^b^
General anaesthesia	0 (0%)	0 (0%)	
Histological chorioamnionitis	5 (63%)	5 (63%)	1.000 ^b^
	IV-paracetamol (*n* = 13)	IV-placebo (*n* = 12)	
**Neonatal** **baseline characteristics**			
Gestational age (weeks)	28^+2^ (27^+0^–29^+6^)	28^+2^ (27^+5^–29^+4^)	0.583 ^a^
Birthweight (g)	1100 (1010–1355)	1190 (955–1385)	0.643 ^a^
Male	7 (54%)	9 (75%)	0.271 ^b^
Apgar score 1 min	7 (4–8)	7 (5–8)	0.803 ^b^
Apgar score 5 min	8 (8–9)	8 (7–9)	0.932 ^b^
Umbilical pH	7.3 (7.1–7.3)	7.3 (7.2–7.3)	0.607 ^a^
Physiological-based cord clamping	4 (31%)	4 (33%)	0.891 ^b^

Missing data comprised 1/9 and 1/9 pregnant women for earlier use of paracetamol during pregnancy. Missing data comprised 1/9 and 1/9 pregnant women for histological chorioamnionitis. Missing data comprised 8/13 and 4/12 infants for umbilical pH. Small for gestational age was defined as a birthweight under the tenth percentile of the corresponding gestational age. ^a^ Mann–Whitney U test. ^b^ Pearson Chi^2^ test. ^c^ Independent-Samples Student’s *t*-test. ^d^ Fisher’s exact test.

**Table 2:** Respiratory and additional physiological parameters in the first 1–10 min after birth.

	IV-Paracetamol (*n* = 13)	IV-Placebo (*n* = 12)	*p*-Value ^1^
**Respiratory** **parameters in the first 10 min after birth**			
Time until start of respiratory support (min)	1:21 ± 0:54	1:35 ± 1:00	0.584 ^a^
Inspiratory tidal volume (mL/kg/breath)	3.72 ± 0.42	3.31 ± 0.41	0.502 ^b^
Respiratory rate (breaths/min)	41 ± 3	34 ± 4	0.200 ^b^
Inspiratory drive (mL/kg/breath/s)	11.8 ± 1.0	8.4 ± 1.3	0.046 ^b^
Inter-breath interval COV (%)	50 ± 4	45 ± 4	0.393 ^b^
Incidence of apnoea (*n*)	0 (0–1)	1 (0–3)	0.403 ^c^
**Additional physiological parameters in the first 10 min after birth**			
HR (beats/min)	137 ± 5	121 ± 5	0.037 ^b^
Time until HR > 100 beats/min (min)	2:10 (1:41–2:25)	2:51 (2:31–3:13)	0.019 ^c^
SpO_2_ (%)	84 ± 3	81 ± 3	0.448 ^b^
Time until SpO_2_ > 80% (min)	2:36 (1:55–3:11)	3:03 (2:07–3:50)	0.211 ^c^
SpO_2_ at 5 min after birth (%)	93 ± 6	83 ± 19	0.120 ^a^
SpO_2_ at 5 min > 80%	13 (100%)	9 (75%)	0.096 ^d^
FiO_2_ (%)	65 ± 7	67 ± 7	0.808 ^b^

Minute volume and tidal volume missing for 1/13 (8%) and 2/12 (17%) infants. Respiratory rate and other respiratory parameters missing for 1/13 (8%) and 1/12 (8%) infants. FiO_2_ data missing for 2/13 (15%) and 2/12 (17%) infants. ^1^ *p*-Values were not corrected for multiple testing or clustering of infants from multiple pregnancies. ^a^ Independent-Samples Student’s *t*-test. ^b^ Linear mixed model with a first-order autoregressive variance covariance matrix and treatment, time and treatment*time as covariates. ^c^ Mann–Whitney U test. ^d^ Fisher’s exact test.

**Table 3:** Respiratory support parameters.

	IV-Paracetamol (*n* = 13)	IV-Placebo (*n* = 12)	Odds Ratio (95% Confidence Interval)	*p*-Value ^1^
**Delivery** **room outcomes**				
Positive pressure ventilation	8 (62%)	6 (50%)	1.60 (0.33–7.85)	0.561 ^a^
Duration of positive pressure ventilation	0:50 (0:00–1:44)	0:18 (0:00–1:39)	-	0.568 ^b^
Intubation	0 (0%)	1 (8%)	-	0.480 ^c^
Caffeine administration	8 (62%)	7 (58%)	1.14 (0.23–5.67)	0.870 ^a^
- Timing of caffeine administration	- 15:20 (11:06–23:11)	- 15:00 (10:40–20:02)	-	
**Neonatal intensive care unit outcomes**				
First rectal temperature measured (°C)	36.6 ± 0.6	36.5 ± 0.6	-	0.830 ^d^
Surfactant administration	8 (62%)	7 (58%)	1.14 (0.23–5.67)	0.870 ^a^
Intubation	5 (39%)	5 (42%)	0.88 (0.18–4.34)	0.870 ^a^

There is no missing data for this table. Duration of positive pressure ventilation was measured in the first 10 min after birth. ^1^ *p*-Values were not corrected for multiple testing or clustering of infants from multiple pregnancies. ^a^ Pearson Chi^2^ test. ^b^ Mann–Whitney U test. ^c^ Fisher’s exact test. ^d^ Independent-Samples Student’s *t*-test.

## Data Availability

The data presented in this study are available on request from the corresponding author due to privacy and ethical restrictions.

## Supplementary Materials

The following supporting information can be downloaded at: https://www.mdpi.com/article/10.3390/children13081061/s1. The Supplemental File contains the exclusion criteria for the study and the resuscitation protocol in the LUMC for premature infants < 31 weeks’ gestation.
