# Supplementary material for: The Effect of Antenatal Paracetamol on Breathing Effort of Premature Infants at Birth: A Feasibility Trial
Source: Children (Basel). 2026 Aug 10;13(8):1061. doi: 10.3390/children13081061 (PMC13510639; doi:10.3390/children13081061)
Supplement: Supplementary file 1 [file children-13-01061-s001.zip › children-4394271-supplementary.pdf]

Exclusion criteria:

- Fetuses with congenital anomalies affecting the heart, lungs, kidneys or liver.
- Pregnant women in whom the use of paracetamol is contraindicated for any reason, consisting of: severe renal impairment, severe hepatic impairment, severe active liver disease (including HELLP-syndrome), a known alcohol addiction and a known hypersensitivity to paracetamol or to any of the excipients in the intravenous formulation.
- Pregnant women that use one of the following medication, which interact with paracetamol: probenecid, salicylamide, rifampicin, isoniazid, barbiturates, tricyclic antidepressants, anti-epileptic medication, zidovudine, oral anticoagulants, metoclopramide, domperidone, cholestyramine, flucloxacillin and imatinib.
- Paracetamol administration required for standard care within 4 hours prior to birth.
- Decision to give palliative care to the neonate.

Resuscitation protocol Leiden University Medical Centre (LUMC)

In the LUMC, respiratory support of the infant is provided by the Neopuff infant T-piece resuscitator (Fisher & Paykel Healthcare, Auckland, New Zealand) with a facemask attached (Neonatal Resuscitation Mask, Fisher & Paykel Healthcare Ltd). The bias gas flow was set to 12 L/min. Respiratory support was initiated with a continuous positive airway pressure set at 5-8 cm H_2O and switched to intermittent positive pressure ventilation during apnoea with a frequency of 30 inflations per minute, a positive end expiratory pressure of 5-8 cm H_2O and a positive inspiratory pressure of 25-30 cm H_2O. Caregivers may also administer caffeine at their discretion. An initial fraction of inspired oxygen of 100% or 50% was used based on the gestation of the infant and titrated according to the 25th percentile of Dawson's nomogram (Dawson JA et al., Pediatrics. 2010 Jun;125(6):e1340-7). Cord clamping occurred between 30-60 seconds after birth and respiratory support was commenced on a standard resuscitation table until April 2024. From April 2024 onward, respiratory support was provided with the same equipment but attached to a purpose-built resuscitation table, the Concord, which was developed at the LUMC to allow for physiological-based cord clamping (clamping of the cord when oxygen saturation ≥85%, fraction of inspired oxygen ≤40% and a heart rate ≥100 beats/minute). Physiological-based cord clamping was only provided to premature infants with vaginal birth for the remainder of the study period. A respiratory function monitor (Advanced Life Diagnostics resuscitation monitor, Weener, Germany) was used to visualize pressures and delivered tidal volumes.
